# PSMA PET/CT-guided salvage elective nodal radiotherapy for nodal metastases after radical prostatectomy: a bi-institutional long-term outcome analysis

**DOI:** 10.1007/s00259-026-08147-z

**Published:** 2026-09-04

**Authors:** Samuel M. Vorbach, Thomas Seppi, Giulia Santo, Irene Virgolini, Ute Ganswindt, Michael Keilholz, Sarah Frederike Brose, Jozefina Casuscelli, Konrad Klimek, Rudolf A. Werner, Nina-Sophie Schmidt-Hegemann, Claus Belka, Christian Trapp, Paul Rogowski

**Affiliations:** 1 Department of Radiation Oncology, Medical University of Innsbruck, Innsbruck, Austria; 2 Department of Nuclear Medicine, Medical University of Innsbruck, Innsbruck, Austria; 3 Department of Radiation Oncology, LMU University Hospital, LMU Medizin, LMU Munich, Munich, Germany; 4 Department of Urology, LMU University Hospital, LMU Medizin, LMU Munich, Munich, Germany; 5 Department of Nuclear Medicine, LMU University Hospital, LMU Medizin, LMU Munich, Munich, Germany; 6 Bavarian Cancer Research Center (BZKF), Munich, Germany; 7 German Cancer Consortium (DKTK), Partner Site Munich, Munich, Germany

**Keywords:** PSMA PET/CT, Prostate cancer, Nodal metastases, Elective nodal radiotherapy, ENRT

## Abstract

**Purpose:**

PSMA PET/CT has transformed the detection of nodal recurrence after radical prostatectomy, yet real-world evidence on long-term outcomes of PSMA PET/CT-guided salvage elective nodal radiotherapy (ENRT) remains limited. This study evaluated oncological outcomes of PSMA PET/CT-guided salvage ENRT in a bi-institutional cohort with extended follow-up.

**Methods:**

We retrospectively analysed 179 patients who received PSMA PET/CT-based ENRT for nodal recurrence after radical prostatectomy at two tertiary centres between 2014 and 2024. Patients with pelvic and/or paraaortic lymph node metastases were included. The primary endpoint was metastasis-free survival (MFS); secondary endpoints included biochemical progression-free survival (BPFS) and overall survival (OS). Survival outcomes were estimated using the Kaplan–Meier method, and prognostic factors were assessed using Cox regression analysis.

**Results:**

Of the 179 patients, 104 (58.1%) had true biochemical recurrence after radical prostatectomy, whereas 75 (41.9%) had PSA persistence. After a median follow-up of 60.3 months, 5-year MFS and BPFS rates were 74.3% and 62.3%, respectively, in patients with true biochemical recurrence, compared with 61.2% and 56.4% in the overall cohort. In multivariable analyses of the entire cohort, concomitant ADT was associated with improved MFS (HR 0.36, *p* < 0.001) and BPFS (HR 0.26, *p* < 0.001), compared with no ADT. PSA persistence after surgery was associated with worse outcomes (MFS: HR 2.72, *p* < 0.001; BPFS: HR 2.09, *p* = 0.005).

**Conclusion:**

PSMA PET/CT-guided salvage ENRT predominantly combined with ADT provides durable long-term disease control in patients with nodal recurrence in routine clinical practice. In patients with true recurrence after radical prostatectomy, the 5-year MFS rate was 74.3%, approaching outcomes reported in prospective trials. However, the absence of systematically collected clinician-assessed toxicity and patient-reported outcomes precludes a reliable assessment of treatment tolerability.

**Supplementary Information:**

The online version contains supplementary material available at https://doi.org/10.1007/s00259-026-08147-z.

## Introduction

Nodal recurrence represents a frequent pattern of recurrence following radical prostatectomy (RP) and occurs in a substantial proportion of patients [1–4]. The optimal management remains controversial for these patients, with available treatment strategies including salvage lymph node dissection, systemic therapy, and radiotherapy-based approaches [5].

In recent years, advances in molecular imaging, particularly prostate-specific membrane antigen positron emission tomography/computed tomography (PSMA PET/CT), have significantly improved the detection of nodal oligorecurrence at low prostate-specific antigen (PSA) levels compared with conventional imaging, thereby enabling more targeted therapeutic approaches [6–8]. Metastasis-directed therapy (MDT), such as stereotactic body radiotherapy (SBRT) to PET-positive lymph nodes or salvage lymphadenectomy, has emerged as an increasingly used strategy in this setting [9, 10].

More recently, the randomised phase II PEACE V-STORM trial demonstrated the superiority of elective nodal radiotherapy (ENRT) compared to an MDT approach in patients with nodal oligorecurrent prostate cancer [11]. In this study, ENRT resulted in significantly improved metastasis-free survival (MFS), with 4-year rates of 76% compared to 63% in the MDT arm, supporting findings from a previous retrospective study by the same group [12]. Notably, all patients received short-term androgen deprivation therapy (ADT) for six months, and PSMA PET imaging was used for staging in 82% of cases.

Despite these encouraging prospective data, evidence regarding PSMA PET/CT-staged salvage ENRT in routine clinical practice remains limited. Most available studies have relied on choline PET/CT or conventional imaging, both of which are inferior to PSMA PET/CT [13]. In particular, real-world outcomes with longer follow-up are still scarce.

Therefore, the aim of this retrospective bi-institutional study was to evaluate oncological outcomes of salvage ENRT in patients with nodal recurrence of prostate cancer in a real-world cohort with extended follow-up.

## Patients and methods

### Study population

This retrospective analysis included patients who underwent PSMA PET/CT-based elective nodal radiotherapy for PSMA PET/CT-positive lymph nodes between 2014 and 2024 at the Department of Radiation Oncology, Medical University of Innsbruck, Austria and the Department of Radiation Oncology, University Hospital, LMU Munich, Germany. Eligible patients had histologically confirmed prostate cancer and underwent PSMA PET/CT during postoperative follow-up because of persistent or rising PSA after RP. PSMA PET/CT was used to localize recurrent or persistent disease and to guide salvage radiotherapy. No maximum number of PSMA PET/CT-positive lymph nodes was applied as an eligibility criterion. Patients who had received previous radiotherapy to the prostate bed were also included. Patients with M1a disease limited to the paraaortic region below the renal arteries were included, whereas patients with M1b/M1c disease were excluded.

### PSMA PET/CT ligand and imaging protocol

Pre-treatment PSMA PET/CT was performed using either [^68^Ga]Ga-PSMA-11 or [^18^F]PSMA-1007. In 15% of patients, [^18^F]PSMA-1007 was used, while the remaining patients underwent imaging with [^68^Ga]Ga-PSMA-11. In Munich, PSMA PET/CT imaging was performed on a Siemens Biograph 64 or GE Discovery 690 scanner, whereas in Innsbruck a GE DMI or GE Discovery 690 scanner was used. For attenuation correction, either a contrast-enhanced diagnostic CT (120 kV, 100–400 mAs with dose modulation) or a low-dose CT (120 kV, 25 mAs) was performed. PSMA PET/CT scans were obtained approximately 60 min after intravenous injection of the respective radiopharmaceutical. Unless contraindicated, patients received 20 mg furosemide at tracer injection to reduce urinary bladder activity and radiation exposure. PET/CT scans were jointly reviewed by a nuclear medicine physician and a radiologist, each with over 5 years of experience. Lesion localization was determined on CT. Assessment of PSMA PET-positive tumour lesion was performed in accordance with EANM guidelines [14].

### Radiotherapy treatment and follow-up

In Innsbruck, patients were treated with three-dimensional conformal radiotherapy (3D-CRT) until 2017, after which volumetric modulated arc therapy (VMAT) was implemented. Daily cone-beam CT was used for image guidance. A normofractionated regimen with a simultaneous integrated boost (SIB) to PSMA PET/CT-positive lesions was delivered. In Munich, all patients were treated with intensity-modulated radiotherapy (IMRT) or volumetric modulated arc therapy (VMAT), delivered in five fractions per week. Image-guided radiotherapy was performed using cone-beam CT 2–5 times per week. Normofractionated or slightly hypofractionated regimens with a SIB to PSMA PET/CT-positive lesions were applied. The prescribed boost dose to PSMA PET/CT-positive lymph nodes was adapted according to the proximity of adjacent organs at risk.

Target delineation for patients with pelvic-only disease followed the Radiation Therapy Oncology Group atlas and, from 2020 onwards, the NRG Oncology consensus, with adjustments based on PSMA PET/CT findings [15, 16]. In patients with paraaortic nodal disease, the elective treatment volume was extended to include the paraaortic lymphatic pathways up to 2 cm cranial to the most cranial PSMA PET/CT-positive lymph node. The prostate bed was routinely included in the treatment volume in patients without previous prostate-bed irradiation. Exceptions were made in selected patients with negative surgical margins in whom a more limited treatment volume was considered appropriate because of advanced age and/or comorbidities. Dose constraints for organs at risk were applied according to institutional standards and were generally based on contemporary RTOG/NRG recommendations, with adaptations according to the prescribed dose and fractionation schedule [17]. ADT was recommended for all patients for 24 to 36 months, with treatment duration individualized according to comorbidities, treatment-related side effects, and patient preference. Patients underwent follow-up with serial PSA assessments, initially every 3 months after radiotherapy and subsequently every 6–12 months according to PSA kinetics. In case of biochemical progression, PSMA PET/CT was performed for restaging.

### Endpoints and statistical analysis

The primary endpoint was MFS, defined as the time from the last day of ENRT to the occurrence of distant metastatic recurrence on imaging or death from any cause. In patients treated for pelvic nodal disease only, the development of new paraaortic or other extrapelvic lymph node metastases (M1a), bone metastases (M1b), visceral metastases (M1c), or death from any cause was considered an MFS event. Pelvic nodal recurrences were not considered MFS events. In patients treated for paraaortic lymph node metastases, new nodal metastases outside the treated elective field, M1b/M1c disease, or death from any cause were considered MFS events. Nodal recurrences within the irradiated elective field were not considered MFS events. Secondary endpoints were biochemical progression-free survival (BPFS) and overall survival (OS). BPFS was defined as the time from the last day of ENRT to biochemical progression or last follow-up. Biochemical progression was defined as a PSA level of ≥ 0.2 ng/mL above the post-radiotherapy nadir. OS was defined as the time from the last day of ENRT to death or last follow-up. Additionally, a subgroup analysis was performed in patients with true biochemical recurrence, excluding those with PSA persistence. Patients without an event were censored at the date of their last available follow-up.

Statistical analysis was performed using SPSS Statistics (V26, IBM Corporation, Armonk, NY, USA), GraphPad Prism (V10, GraphPad Software Inc., San Diego, CA, USA), and R (V4.6.1, R Foundation for Statistical Computing, Vienna, Austria). Descriptive analysis was used to summarise key patient and treatment characteristics. The Kaplan-Meier method was used to calculate MFS, BPFS and OS. The Cox proportional hazards model was used for univariate and multivariate analyses of prognostic factors. The proportional hazards assumption was assessed using scaled Schoenfeld residuals, and no significant violations were detected. The PSA before ENRT was dichotomised at 1.0 ng/mL based on the threshold used in the analysis of the PEACE V–STORM trial [11]. Variables with a p value < 0.1 in univariate analysis were entered into the multivariate model. Multivariate analyses were performed as complete-case analyses and included only patients with available data for all covariates entered into the respective model. Stepwise backward elimination was subsequently applied using a retention threshold of *p* < 0.10. A p value < 0.05 was considered statistically significant.

## Results

A total of 179 patients underwent PSMA PET/CT-based ENRT for PSMA PET/CT-positive lymph nodes after RP. Median follow-up was 60.3 months (range, 12.9-135.9 months). Patient characteristics are shown in Table 1. Median age at ENRT was 71 years (range, 48–82 years). Most patients had initial tumour stage ≥pT3 (73.2%) and were pN0 (64.2%). Overall, 43.6% had positive surgical margins and 50.9% had an International Society of Urological Pathology (ISUP) score of 4 or 5. The median interval between RP and identification of PSMA PET/CT-positive lymph nodes was 13.0 months. The median PSA level at the time of PSMA PET/CT was 0.86 ng/mL (range, 0.06–40.13 ng/mL), with 23 of 179 patients (12.8%) having a PSA level above 5 ng/mL. PSMA PET/CT showed one, two, or ≥ 3 positive lymph nodes in 47.5%, 26.8%, and 25.7% of patients, respectively. Most patients had pelvic lymph node metastases only (89.9%), whereas 18 patients (10.1%) had paraaortic lymph node metastases, with or without additional pelvic involvement.

**Table 1:** Patient characteristics

Characteristic	Value or no. (%)
Total No. of patients	179
Age at the time of sENRT (years)	
Median (range)	71 (48–82)
Initial tumour stage	
pT2a	5 (2.8%)
pT2b	5 (2.8%)
pT2c	38 (21.2%)
pT3a	58 (32.4%)
pT3b	68 (38.0%)
pT4	5 (2.8%)
Initial nodal stage	
pN0	115 (64.2%)
pN1	53 (29.6%)
pNx/cN0	9 (5.0%)
Unknown	2 (1.1%)
Pelvic lymph node dissection at RP	
Yes	169 (94.4%)
No	10 (5.6%)
Number of lymph nodes removed	
Median (range)	12 (3–45)
Positive surgical margins	78 (43.6%)
ISUP score	
1	4 (2.2%)
2	32 (17.9%)
3	52 (29.1%)
4	32 (17.9%)
5	59 (33.0%)
PSA at initial diagnosis (ng/mL)	
Median (range)	10.50 (1.60–332.00)
PSA post RP (ng/mL)	
Median (range)	0.10 (0.00–40.13)
Patients with BCR/BCP	104 (58.1%) / 75 (41.9%)
RT to prostate bed before sENRT for prior BCR	24 (13.4%)
Time between RP and diagnosis of nodal recurrence (months)	
Median (range)	13.0 (1–175)
PSA at PSMA PET/CT	
Median (range)	0.86 (0.06–40.13)
Number of PSMA PET/CT-positive lymph nodes	
1	85 (47.5%)
2	48 (26.8%)
≥ 3	46 (25.7%)
Localisation of PSMA PET/CT-positive lymph nodes per patient	
Pelvic only	161 (89.9%)
Paraaortic (± pelvic)	18 (10.1%)
Patients with local recurrence on PSMA PET/CT	41 (22.9%)

Abbreviation: *sENRT* salvage elective nodal radiotherapy, *RP* radical prostatectomy, *ISUP* International Society of Urological Pathology, *PSA* prostate-specific antigen, *BCR* biochemical recurrence, *BCP* biochemical persistence

Treatment characteristics are summarized in Table 2. Median EQD2 doses (α/β = 1.5 Gy) were 47.5 Gy (range, 41.6–50.9 Gy) to the elective nodal regions and 66.0 Gy (range, 57.5–72.0 Gy) to the prostate bed. In patients with local recurrence, the median dose was 70.0 Gy (range, 66.0–76.8 Gy), while PSMA PET-positive lymph nodes received a median dose of 62.3 Gy (range, 51.7–66.0 Gy). Concomitant ADT was administered in 83.8% of patients, while 16.2% received no ADT. Among patients receiving ADT, treatment duration was ≤ 12 months in 31.3%, and > 12 months in 68.0%.

**Table 2:** Treatment characteristics

Characteristic	Value or no. (%)
ADT	
No ADT	29 (16.2%)
Concomitant ADT	150 (83.8%)
ADT Duration	
No ADT	29 (16.2%)
≤ 12 months	47 (26.3%)
> 12 months	102 (57.0%)
Unknown	1 (0.6%)
Completion of initial ADT at last follow-up	
Yes	169 (94.4%)
No	9 (5.0%)
Unknown	1 (0.6%)
ADT-free time in patients without ADT at last follow-up (months)	
Median (range)	50.5 (1–134)
Dose to prostate bed EQD2_1.5_ (Gy)	
Median (range)	66.0 (57.5–72.0)
Dose to local recurrence EQD2_1.5_ (Gy)	
Median (range)	70.0 (66.0–76.8)
Dose to elective lymph node regions EQD2_1.5_ (Gy)	
Median (range)	47.5 (41.6–50.9)
Dose to PSMA PET/CT-positive lymph nodes EQD2_1.5_ (Gy)	
Median (range)	62.3 (51.7–66.0)

Abbreviation: *ADT* androgen deprivation therapy

**Fig. 1:**
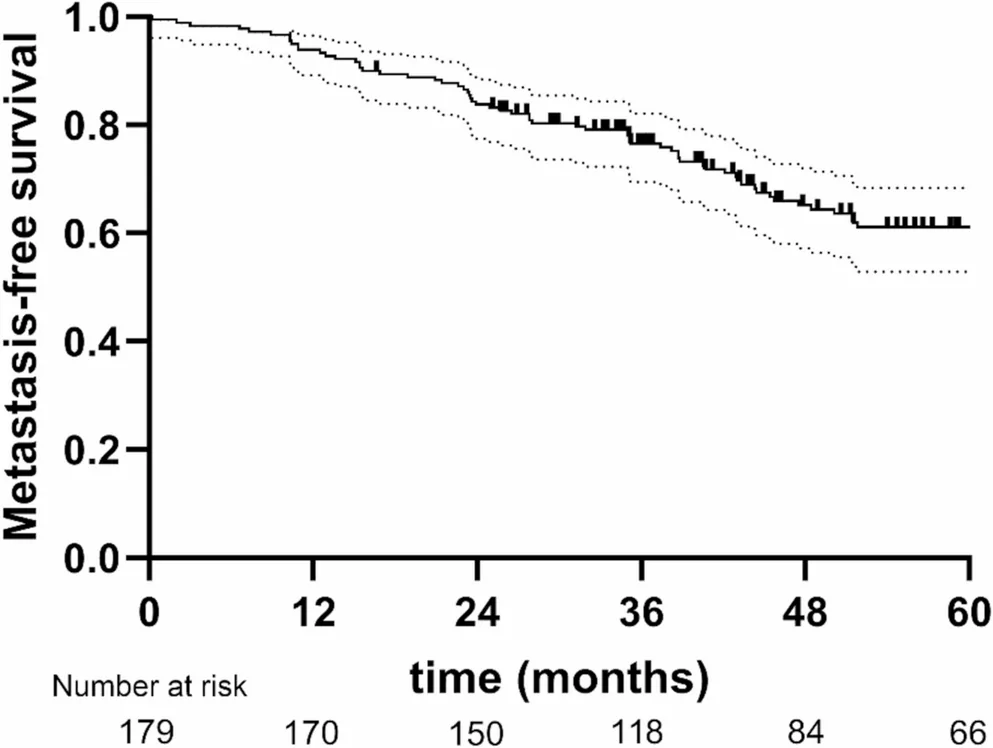
Kaplan Meier curve showing metastasis-free survival (dotted lines: 95% confidence interval)

The 3-, 4- and 5-year MFS rates were 76.5% (95% CI: 69.5–82.1), 65.2% (95% CI: 57.2–72.1) and 61.2% (95% CI: 52.9–68.5), respectively (Fig. 1). In univariate analysis, paraaortic lymph node involvement (vs. pelvic lymph node metastases only, HR = 2.20, 95% CI: 1.18–4.08, *p* = 0.013, Table 3), concomitant ADT (vs. no ADT, HR = 0.55, 95% CI: 0.31–0.97, *p* = 0.041), PSA persistence after RP (vs. recurrence, HR = 2.20, 95% CI: 1.57-4.00, *p* < 0.001), and PSA ≥ 1.0 ng/mL before ENRT (vs. < 1.0 ng/mL, HR = 1.74, 95% CI: 1.10–2.75, *p* = 0.017) were significantly associated with MFS. In multivariate analysis, concomitant ADT (vs. no ADT, HR = 0.36, 95% CI: 0.20–0.66, *p* < 0.001) was associated with improved MFS, whereas PSA persistence after RP remained associated with worse MFS (vs. recurrence, HR = 2.72, 95% CI: 1.64–4.53, *p* < 0.001). In addition, paraaortic lymph node involvement showed a trend toward worse MFS compared with pelvic lymph node metastases only, but did not reach statistical significance (HR = 1.74, 95% CI: 0.91–3.34, *p* = 0.094).

**Table 3:** Univariate and multivariate analysis of risk factors related to MFS

Factor	*N*	Univariate Hazard Ratio for MFS		Multivariate Hazard Ratio for MFS	
		HR (95% CI)	*p* Value	HR (95% CI)	*p* Value
PSMA PET/CT tracer					
[^68^Ga]Ga-PSMA-11	149	1 (reference)			
[^18^F]PSMA-1007	27	1.06 (0.56–2.03)	0.854		
Number of lymph node metastases					
1	85	1 (reference)			
≥ 2	94	1.27 (0.80–2.02)	0.310		
Lymph node localisation					
Pelvic	161	1 (reference)			
Paraaortic (± pelvic)	18	2.20 (1.18–4.08)	0.013	1.74 (0.91–3.34)	0.094
Concomitant ADT					
No	29	1 (reference)			
Yes	149	0.55 (0.31–0.97)	0.041	0.36 (0.20–0.66)	< 0.001
PSA persistence vs. recurrence					
Recurrence	104	1 (reference)			
Persistence	75	2.20 (1.57–4.00)	< 0.001	2.72 (1.64–4.53)	< 0.001
PSA before sENRT					
< 1.0 ng/mL	98	1 (reference)			
≥ 1.0 ng/mL	61	1.74 (1.10–2.75)	0.017		
Local recurrence					
No local recurrence	138	1 (reference)			
Local recurrence	41	1.15 (0.68–1.96)	0.604		

Abbreviations: *CI* confidence interval, *PSMA PET/CT* prostate-specific membrane antigen positron emission tomography/computed tomography, *ADT* androgen deprivation therapy, *PSA* prostate-specific antigen, *sENRT* salvage elective nodal radiotherapy

**Fig. 2:**
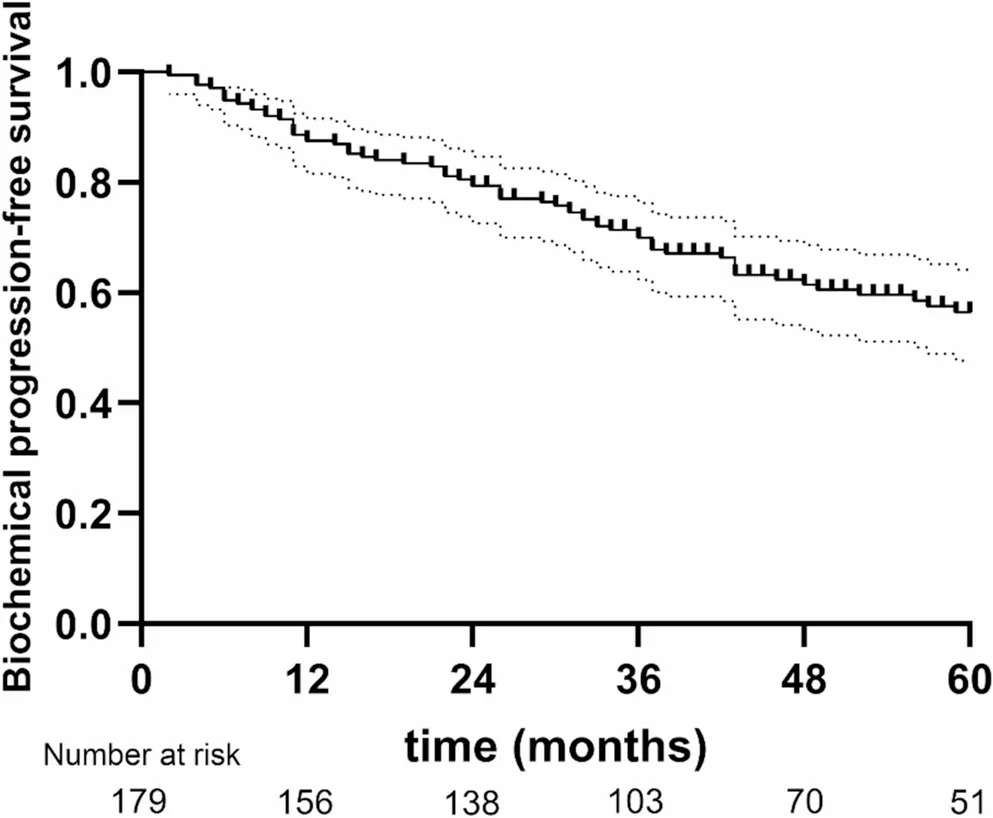
Kaplan Meier curve showing biochemical progression-free survival (dotted lines: 95% confidence interval)

The 3-, 4- and 5-year BPFS rates were 70.0% (95% CI: 62.4–76.3), 61.5% (95% CI: 53.3–68.7) and 56.4% (95% CI: 47.7–64.2), respectively (Fig. 2). In univariate analysis, concomitant ADT (vs. no ADT, HR = 0.42, 95% CI: 0.24–0.73, *p* = 0.002, Table 4), PSA persistence (vs. recurrence, HR = 1.74, 95% CI: 1.17–2.76, *p* = 0.018), and a PSA of ≥ 1.0 ng/mL before ENRT (vs. < 1.0 ng/mL, HR = 1.74, 95% CI: 1.10–2.75, *p* = 0.017) were significantly associated with BPFS, while lymph node localisation (paraaortic ± pelvic vs. pelvic, HR = 1.89, 95% CI 0.97–3.69, *p* = 0.061) showed a trend toward an association with BPFS. In multivariate analysis, concomitant ADT (vs. no ADT, HR = 0.26, 95% CI: 0.14–0.49, *p* < 0.001) was associated with improved BPFS, whereas PSA persistence after RP (vs. recurrence, HR = 2.09, 95% CI: 1.23–3.48, *p* = 0.005) and PSA of ≥ 1.0 ng/mL before ENRT (vs. < 1.0 ng/mL, HR = 1.76, 95% CI: 1.08–2.89, *p* = 0.025) were associated with worse BPFS. In an exploratory subgroup analysis, the 3-, 4-, and 5-year MFS and BPFS rates were calculated according to ADT exposure and duration (no ADT, ADT ≤ 12 months, and ADT > 12 months) and are presented in Supplementary Table 3.

Overall, 20 patients died during the follow-up period, of whom 3 had documented metastatic prostate cancer before death. The 3-, 4-, and 5-year OS rates were 96.5% (95% CI: 92.3–98.4), 92.3% (95% CI: 86.8–95.6), and 90.6% (95% CI: 84.5–94.3), respectively.

Among patients with true biochemical recurrence after RP (104/179), excluding those with biochemical persistence, the 3-, 4- and 5-year MFS rates were 87.3% (95% CI: 79.1–92.4), 76.0% (95% CI: 62.5–83.7) and 74.3% (95% CI: 63.1–82.6), respectively. The 3-, 4- and 5-year BPFS rates of this cohort were 78.5% (95% CI: 68.9–85.5), 69.2% (95% CI: 57.8–78.1) and 62.3% (95% CI: 49.4–72.8), respectively. Kaplan–Meier curves directly comparing patients with true biochemical recurrence and those with PSA persistence are provided for MFS and BPFS in Supplementary Figs. 1 and 2, respectively. No significant prognostic factors for MFS were identified in this subgroup (Supplementary Table 1). In multivariable analysis of BPFS, the presence of ≥ 2 lymph node metastases was independently associated with worse BPFS (HR = 2.15, 95% CI: 1.06–4.36, *p* = 0.035), whereas concomitant ADT was independently associated with improved BPFS compared with no ADT (HR = 0.34, 95% CI: 0.16–0.74, *p* = 0.007; Supplementary Table 2). The OS rates were 95.0% (95% CI: 88.4–97.9) at 3 years, 90.8% (95% CI: 82.3–95.4) at 4 years, and 89.2% (95% CI: 79.9–94.4) at 5 years.

**Table 4:** Univariate and multivariate analysis of risk factors related to BPFS

Factor	*N*	Univariate Hazard Ratio for BPFS		Multivariate Hazard Ratio for BPFS	
		HR (95% CI)	*p* Value	HR (95% CI)	*p* Value
PSMA PET/CT tracer					
[^68^Ga]Ga-PSMA-11	149	1 (reference)			
[^18^F]PSMA-1007	27	1.42 (0.79–2.55)	0.239		
Number of lymph node metastases					
1	85	1 (reference)			
≥ 2	94	1.12 (0.71–1.78)	0.621		
Lymph node localisation					
Pelvic	161	1 (reference)			
Paraaortic (± pelvic)	18	1.89 (0.97–3.69)	0.061		
Concomitant ADT					
No	29	1 (reference)		1 (reference)	
Yes	148	0.42 (0.24–0.73)	0.002	0.26 (0.14–0.49)	< 0.001
PSA persistence vs. recurrence					
Recurrence	104	1 (reference)		1 (reference)	
Persistence	75	1.74 (1.17–2.76)	0.018	2.09 (1.23–3.48)	0.005
PSA before sENRT					
< 1.0 ng/mL	98	1 (reference)		1 (reference)	
≥ 1.0 ng/mL	61	1.74 (1.10–2.75)	0.017	1.76 (1.08–2.89)	0.025
Local recurrence					
No local recurrence	138	1 (reference)			
Local recurrence	41	0.88 (0.50–1.56)	0.662		

Abbreviations: *CI* confidence interval, *PSMA PET/CT* prostate-specific membrane antigen positron emission tomography/computed tomography, *ADT* androgen deprivation therapy, *PSA* prostate-specific antigen, *sENRT* salvage elective nodal radiotherapy

The locations of relapse are shown in Table 5. Biochemical relapse alone, defined as a rise in PSA without evidence of recurrent disease on subsequent PSMA PET/CT imaging, was observed in 19 patients (10.6%) of the overall study population. Distant metastases represented the most frequent pattern of relapse. Among distant metastatic lesions, bone metastases (M1b) were the most common, occurring in 20.7% of the overall study population, followed by distant lymph node metastases (M1a) in 14.0% and organ metastases (M1c) in 3.4%. Multiple anatomical sites of relapse per patient were possible. Among the 54 patients with imaging-confirmed recurrence, 4 (7.4%) developed an isolated in-field recurrence, 39 (72.2%) developed an isolated out-of-field recurrence, and 11 (20.4%) experienced both in-field and out-of-field recurrence during the course of their disease.

**Table 5:** Location of relapse

Location of relapse	No. (%)
Biochemical relapse only	19 (10.6%)
Prostate bed	3 (1.7%)
Pelvic nodes	7 (3.9%)
Distant metastases	
Lymph nodes (M1a)	25 (14.0%)
Bone (M1b)	37 (20.7%)
Organs (M1c)	6 (3.4%)

Multiple metastatic sites per patient were possible

## Discussion

In this bi-institutional study, we report long-term real-world outcomes of PSMA PET/CT-guided salvage ENRT in patients with nodal recurrent prostate cancer after RP. With a median follow-up exceeding five years, our data demonstrate that PSMA PET/CT-guided ENRT predominantly combined with ADT achieves durable long-term disease control in routine clinical practice, with 5-year MFS above 60%. These findings support ENRT as an effective treatment strategy in the PSMA PET era, particularly when combined with ADT.

Our results align with the randomised phase II PEACE V–STORM trial, which demonstrated the superiority of ENRT over MDT in nodal oligorecurrent prostate cancer [11]. Notably, outcomes in our cohort were somewhat lower despite similar treatment principles. This difference is likely explained by a less selected, higher-risk population in routine clinical practice, characterised by a higher proportion of high-grade disease (ISUP ≥ 4), a greater nodal burden, inclusion of patients with biochemical persistence, and the presence of paraaortic metastases. In addition, ADT use in our cohort was heterogeneous and frequently prolonged, in contrast to the standardized short-course ADT applied in PEACE V. While these comparisons provide important clinical context, our findings should be interpreted as long-term real-world outcome data for PSMA PET/CT-guided salvage ENRT rather than as comparative evidence in favour of this approach over alternative local treatment strategies.

Biochemical persistence after RP emerged as a strong and independent predictor of adverse outcomes in our cohort. This subgroup is known to have an unfavourable prognosis [11], likely reflecting occult regional and/or distant metastatic disease already present at the time of surgery. When restricting the analysis to patients with true biochemical recurrence, outcomes were markedly improved, with 3-, 4-, and 5-year MFS rates of 87%, 76%, and 74%, respectively, closely matching the results of PEACE V. Within this more homogeneous subgroup, no independent prognostic factors for MFS were identified, whereas a higher nodal burden (≥ 2 PSMA PET-positive lymph nodes) remained independently associated with worse BPFS. These findings should be interpreted cautiously given the substantially reduced sample size and the exploratory nature of the subgroup analysis.

Approximately 10% of patients in our cohort presented with paraaortic lymph node metastases (M1a), whereas PEACE V excluded patients with nodal disease above the aortic bifurcation. Paraaortic involvement represents a more advanced disease stage and likely reflects early systemic dissemination. In our multivariable analysis, paraaortic involvement showed a trend toward worse MFS, although this did not reach statistical significance (HR 1.74, *p* = 0.094).

The role of ADT warrants particular attention. In our cohort, concomitant ADT was strongly associated with improved BPFS and MFS, with effect sizes that exceed those reported in postoperative radiotherapy meta-analyses [18]. This likely reflects the higher disease burden in patients with PSMA PET-detected nodal metastases, in whom combined modality treatment may be required to achieve durable disease control. However, the effect of ADT duration cannot be reliably assessed from our data. Because treatment duration was defined retrospectively according to the therapy actually administered rather than the intended duration at treatment initiation, analyses based on ADT duration would be susceptible to time-dependent and immortal-time bias [19]. We therefore restricted the Cox regression analyses to concomitant ADT as a binary variable (present vs. absent), reflecting the treatment decision made at the time of ENRT, and reported ADT duration descriptively only. The optimal duration of ADT in patients undergoing salvage ENRT for nodal recurrence remains uncertain and should be addressed in prospective studies.

Pre-treatment PSA level was associated with BPFS, with improved outcomes observed in patients treated at PSA < 1.0 ng/mL, supporting early intervention in the oligorecurrent setting. However, PSA did not maintain independent significance for MFS, suggesting that its prognostic value may be outweighed by other factors such as disease biology and extent of nodal involvement.

When analysing patterns of failure, a higher proportion of distant metastases was observed in our cohort compared to PEACE V, consistent with the higher-risk profile of our patients. Conversely, rates of prostate bed recurrence were lower. This may be explained by differences in treatment strategies: in our cohort, the prostate bed was routinely included in the RT volume in patients without prior RT, whereas in PEACE V, prostate bed RT was administered only in selected high-risk patients and in just over half of the cohort. Notably, prostate bed RT was a major contributor to genitourinary and gastrointestinal toxicity in PEACE V, highlighting the need for careful patient selection and further investigation of optimal target volumes.

The spatial pattern of recurrence provides further insight into the potential mechanisms of treatment failure. Among patients with imaging-confirmed recurrence, isolated in-field failure occurred in only 7.4%, whereas 72.2% developed isolated out-of-field recurrence and 20.4% experienced both in-field and out-of-field recurrence during the course of their disease. Thus, most patients with an in-field recurrence also developed disease outside the irradiated volume. This pattern suggests that inadequate target coverage was unlikely to be the predominant cause of treatment failure. Instead, the predominance of out-of-field and distant progression may reflect occult micrometastatic disease below the detection threshold of PSMA PET/CT or more aggressive systemic disease biology. Previous pattern-of-failure analyses after PSMA PET-guided radiotherapy have likewise reported predominantly out-of-field progression and relatively uncommon true in-field recurrence [20]. Although PSMA PET/CT provides superior sensitivity compared with conventional imaging, its negative predictive value for nodal disease remains imperfect [7, 21]. Consequently, a negative PSMA PET/CT does not exclude occult metastases, particularly in patients with high-risk clinical features or higher PSA levels at recurrence. The predominance of out-of-field recurrence in our cohort, including a substantial proportion of bone metastases, may therefore reflect occult disease beyond the electively irradiated nodal regions or subsequent systemic dissemination.

Compared with choline PET/CT-guided ENRT studies, outcomes in our study appear more favourable. Prospective data, including the GETUG-P07–OLIGOPELVIS trial, as well as long-term series, have reported lower biochemical and metastasis-free survival rates [22, 23]. This difference may be attributable to the superior sensitivity and specificity of PSMA PET/CT, enabling more accurate staging and improved target volume delineation. However, besides the different tracers used, these studies also differed in terms of inclusion criteria and ADT duration.

Two ongoing phase III trials are currently evaluating the optimal treatment strategy for patients with oligorecurrent pelvic lymph node metastases. The GETUG-P12–OLIGOPELVIS 2 trial is investigating the addition of ENRT to 6 months of ADT and has recently reported favourable early toxicity data, while survival outcomes are still pending [24]. The POINTER-PC trial is comparing SBRT to involved nodes with ENRT delivered in either 5 or 20 fractions [25]. These studies are expected to provide further evidence regarding the optimal management of this patient population.

Our study has several limitations. The retrospective design introduces potential selection bias and limits causal inference. Treatment heterogeneity, including differences in ADT use and duration, treatment centre, treatment era, radiotherapy technique, and the inclusion of patients with biochemical persistence and paraaortic disease, may have influenced the observed outcomes. At the same time, the inclusion of these patient subgroups and the variability in ADT duration reflect a real-world cohort encountered in routine clinical practice and thereby enhance the generalizability of our findings. In addition, differences in tracer type and imaging protocols between institutions may have influenced detection rates and staging accuracy. The quality and completeness of the retrospectively collected data were insufficient to allow a reliable analysis of acute and late genitourinary and gastrointestinal toxicity. Furthermore, health-related quality of life was not systematically assessed using validated patient-reported outcome measures. The exact cause of death could not be reliably established in all patients because of the retrospective study design. Consequently, prostate cancer-specific mortality could not be assessed reliably. In addition, competing-risk analyses were not performed for BPFS, and death without prior biochemical progression was therefore not explicitly accounted for as a competing event. Furthermore, the multivariable Cox regression models were based on data-driven variable selection using univariate screening and stepwise backward elimination, which may increase the risk of model instability and overfitting. The identified independent associations should therefore be regarded as exploratory and require validation in independent cohorts.

## Conclusion

PSMA PET/CT-guided salvage ENRT predominantly combined with ADT achieves durable long-term disease control in patients with nodal recurrent prostate cancer in a real-world setting, with outcomes approaching those of prospective trials despite a higher-risk population. However, the absence of systematically collected clinician-assessed toxicity and patient-reported outcomes precludes a reliable assessment of treatment tolerability. Prospective phase III trials are warranted to further refine patient selection, optimize the integration of systemic therapy, and define the optimal treatment strategy for patients with PSMA PET/CT-detected nodal recurrence.

## Supplementary Information

Below is the link to the electronic supplementary material.

Supplementary Material 1

Supplementary Material 2

Supplementary Material 3

Supplementary Material 4

## Data Availability

The datasets analysed in this study are available from the corresponding author upon reasonable request.
