# Supplementary material for: PSMA PET/CT-guided salvage elective nodal radiotherapy for nodal metastases after radical prostatectomy: a bi-institutional long-term outcome analysis
Source: Eur J Nucl Med Mol Imaging. 2026 Sep 4;53(13):8478–87. doi: 10.1007/s00259-026-08147-z (PMC13727915; doi:10.1007/s00259-026-08147-z)
Supplement: Supplementary file 2 [file 259_2026_8147_MOESM2_ESM.docx]

**Supplementary Table 2** Univariate and multivariate analysis of risk factors related to BPFS in patients with true biochemical recurrence

| **Factor** | **N** | **Univariate Hazard Ratio for BPFS** | | **Multivariate Hazard Ratio for BPFS** | |
| --- | --- | --- | --- | --- | --- |
|  |  | **HR (95% CI)** | ***p* Value** | **HR (95% CI)** | ***p* Value** |
| **PSMA-PET/CT tracer**  [^68^Ga]Ga-PSMA-11  [^18^F]PSMA-1007 | 92  12 | 1 (reference)  2.07 (0.85 - 5.07) | 0.110 |  |  |
| **Number of lymph node metastases**  1  ≥ 2 | 63  41 | 1 (reference)  1.86 (0.93 - 3.73) | 0.080 | 1 (reference)  2.15 (1.06 - 4.36) | **0.035** |
| **Lymph node localisation**  Pelvic  Paraaortic (± pelvic) | 99  5 | 1 (reference)  1.98 (0.47 - 8.39) | 0.355 |  |  |
| **Concomitant ADT**  No  Yes | 21  83 | 1 (reference)  0.40 (0.19 – 0.85) | **0.017** | 1 (reference)  0.34 (0.16 - 0.74) | **0.007** |
| **PSA before sENRT**  < 1.0 ng/ml  ≥ 1.0 ng/ml | 71  33 | 1 (reference)  1.59 (0.77 - 3.23) | 0.196 |  |  |
| **Local recurrence**  No local recurrence  Local recurrence | 81  23 | 1 (reference)  0.45 (0.16 - 1.27) | 0.132 |  |  |

*Abbreviations*: *CI* confidence interval; *PSMA-PET/CT* prostate-specific membrane antigen positron emission tomography/computed tomography; *ADT* androgen deprivation therapy; *PSA* prostate-specific antigen; *sENRT* salvage elective nodal radiotherapy
