# Supplementary material for: PSMA PET/CT-guided salvage elective nodal radiotherapy for nodal metastases after radical prostatectomy: a bi-institutional long-term outcome analysis
Source: Eur J Nucl Med Mol Imaging. 2026 Sep 4;53(13):8478–87. doi: 10.1007/s00259-026-08147-z (PMC13727915; doi:10.1007/s00259-026-08147-z)
Supplement: Supplementary file 3 [file 259_2026_8147_MOESM3_ESM.docx]

|  |  | **Metastasis-free survival, %** | | | **Biochemical progression-free survival, %** | | |
| --- | --- | --- | --- | --- | --- | --- | --- |
| **ADT exposure and duration** | **N** | 3-year, (95% CI) | 4-year (95% CI) | 5-year (95% CI) | 3-year (95% CI) | 4-year (95% CI) | 5-year (95% CI) |
| No ADT | 29 | 61.7 (41.2 - 76.7) | 56.9 (36.4 - 73.0) | 56.9 (36.4 - 73.0) | 50.5 (30.1 - 67.8) | 32.7 (14.0 - 53.0) | 32.7 (14.0 - 53.0) |
| ≤ 12 months | 47 | 69.9 (54.5 - 80.9) | 60.9 (45.3 - 73.3) | 55.8 (40.1 - 68.9) | 56.6 (41.0 - 69.5) | 54.2 (38.8 - 67.2) | 54.2 (38.8 - 67.2) |
| > 12 months | 102 | 84.0 (75.2 - 89.9) | 69.7 (58.7 - 78.3) | 63.6 (52.5 - 72.8) | 81.1(71.6 - 88.4) | 71.5 (60.5 - 79.5) | 62.1 (46.9 - 72.3) |

*Abbreviations*: *CI* confidence interval; *ADT* androgen deprivation therapy
