# Supplementary material for: PSMA PET/CT-guided salvage elective nodal radiotherapy for nodal metastases after radical prostatectomy: a bi-institutional long-term outcome analysis
Source: Eur J Nucl Med Mol Imaging. 2026 Sep 4;53(13):8478–87. doi: 10.1007/s00259-026-08147-z (PMC13727915; doi:10.1007/s00259-026-08147-z)
Supplement: Supplementary file 4 [file 259_2026_8147_MOESM4_ESM.docx]

**Supplementary Figure 1** Metastasis-free survival in patients with true biochemical recurrence versus PSA persistence after radical prostatectomy. Kaplan–Meier curves showing metastasis-free survival in patients with true biochemical recurrence and PSA persistence. Dotted lines indicate 95% confidence intervals. Survival distributions were compared using the log-rank test. The numbers of MFS events were 28 in patients with true biochemical recurrence and 48 in patients with PSA persistence.

**Supplementary Figure 2** Biochemical progression-free survival in patients with true biochemical recurrence versus PSA persistence after radical prostatectomy. Kaplan–Meier curves showing biochemical progression-free survival in patients with true biochemical recurrence and PSA persistence. Dotted lines indicate 95% confidence intervals. Survival distributions were compared using the log-rank test. Thirty-two patients with true biochemical recurrence and 41 patients with PSA persistence experienced biochemical progression.
